# Relationship Between Knowledge Base and Innovation-Driven Growth: Moderated by Organizational Character

**DOI:** 10.3389/fpsyg.2021.663317

**Published:** 2021-05-25

**Authors:** Dengke Yu, Hongling Yan

**Affiliations:** School of Management, Nanchang University, Nanchang, China

**Keywords:** innovation-driven growth, knowledge base, technological innovation, business model innovation, organizational character

## Abstract

**Purpose:** On the background of innovation-driven growth strategy of the Chinese government, this study aims to explore the impact of the knowledge base on innovation-driven growth of a firm, which is moderated by organizational character.

**Design/methodology/approach:** Based on the data of 965 Chinese listed companies, some hypotheses were tested using the method of hierarchical regression analysis.

**Findings:** Organizational growth relies on both technological and business model innovations and their interactive effect. Knowledge base, both breadth and depth, makes a positive impact on the innovation-driven growth of an enterprise. In the impacting mechanism, an explicit organizational character not only has direct positive effects on business model innovation, it also strengthens the effect of knowledge breadth on business model innovation. On the contrary, an implicit organizational character is not significantly related to innovation.

**Research limitations/implications:** In order to achieve growth, enterprises are suggested to adopt such dual innovation strategy, led by technological innovation and supplemented with business model innovation, which is supported by the integrated management of intangible resources, deep and broad knowledge, and explicit organizational character.

**Originality/value:** A new theoretical framework of organizational innovation-driven growth was proposed. The realization paths of innovation-driven growth were explored. The idea of collaborative governance between the knowledge base and organizational character was raised.

## Introduction

In the past three decades, China has developed from a lagging to an emerging economy (Wang et al., 2020). However, nowadays, when bonuses brought by the reform and opening-up policy and population increase are fading away, the Chinese government is eager to seek a new engine for economic growth. Under such circumstances, following the trend of global economic development, the Chinese government proposed the national strategy of innovation-driven growth (Yuan et al., 2018). In order to implement the strategy, China adopted a “mass entrepreneurship and innovation” policy (Wu and Li, 2019). Supported by the policy, over 40 million Chinese small and medium-sized enterprises (SMEs), which account for 99% of Chinese firms, have become the key player in innovation-driven growth (China’s National Bureau of Statistics, 2020).

After examining the development practices of Chinese SMEs, which have boomed in recent years, we found that most of them followed two paths to grow. First, the endogenous growth path, from knowledge resources to organizational performance and sustainable growth, mediated by technological innovation, has been followed by many knowledge-intensive organizations (Huarng et al., 2020; Xiao and Yu, 2020). Second, the exogenous growth path, which focuses on the creation of new business models through integration and utilization of external resources, has been widely introduced in Chinese emerging enterprises for the realization of organizational performance and achievement of competitive advantage (Li and Yu, 2018; An et al., 2021). However, because of the practical examination mentioned above, more academic explorations are urgently required to provide evidence for theoretical development.

In fact, we have reported the above-mentioned development logic by empirical analyses in several literature published by the research team of this study. For instance, Li and Yu (2018) indicated the important role of the combination of technological and business model innovations, and explored the paths to dual innovation. Furthermore, Xiao and Yu (2020) assessed the effects of technological and business model innovations on sustainable competitive advantage. In addition, similar to the setting of this study, knowledge capital and organizational character in the two studies mentioned above were regarded as antecedent enablers of innovation.

In spite of the previous studies, some gaps still exist in the development of an innovation-driven theory. First, in the study published by Li and Yu (2018), the transformation from knowledge capital to organizational character and the impact of technological innovation on business model innovation were assessed from a linear perspective. However, the effects of the combination of knowledge capital and organizational character and technological and business model innovations were neglected. Second, prior studies have focused on the positive influence of knowledge stock, but, from a structural perspective, the discussion of the roles of knowledge base in innovation-driven growth is relatively rare. Third, the paths to sustainable competitive advantage have been explored by Xiao and Yu (2020), but they cannot directly link these paths to the realization of innovation-driven growth of a firm.

In order to close the gaps, the study paid attention to answering the following two research questions:

RQ1. How does a knowledge base facilitate the innovation-driven growth of an enterprise?

RQ2. What is the role of an organizational character in the relationship between the knowledge base and the innovation-driven growth?

The structure of this study is organized as follows. In section Literature Review, the existing studies were reviewed. In section Development of Hypotheses, research hypotheses and a theoretical model were developed. In section Methodology, research procedures and methods were described. Then, in section Results, we tested the hypotheses and analyzed the results. Finally, in section Conclusions, Implications, and Limitations, findings and implications were concluded.

## Literature Review

### Dual Innovation for Growth of Firm

Technological innovation has been proposed as the most important strategy for the sustainable growth of a firm by many scholars for a long time (Fang et al., 2020; Fiorentino et al., 2020; Dalgic and Fazlioglu, 2021; Wan and Zhang, 2021). First, technological innovation would improve the technical skill of a firm and then continuously upgrade its products and services (Shi et al., 2018). Second, technological innovation increases the adaptive capability that helps a firm to meet the challenges from rapid changes in industrial technology (Roy and Sarkar, 2016; Byun et al., 2018).

In addition to technological innovation, business model innovation is also essential to the growth of the firm. First, it may create more resource combinations and product mixtures, which may help firms to better meet the diversified demands in turbulent markets (Rosca et al., 2017; Snihur and Wiklund, 2019). Second, business model innovation may change the manufacturing process, commercial channel, and value proposition of a firm, which may lead to a high efficiency and a strong capability to add value (Yi et al., 2020; Sun et al., 2021; Sundstrom et al., 2021).

Considering the importance of balance and complementarity of technological and business model innovations, Liu et al. (2019) put forward a new type of dual innovation system, which emphasizes their integration based on the ambidexterity theory. Similarly, some studies have discussed the relationship between technological and business model innovations and have assessed the effects of their combination (Hu, 2014; Li and Yu, 2018; Rantala et al., 2018; Xiao and Yu, 2020).

### Knowledge Base for Innovation of Firm

Knowledge is vital for innovation of the firm. First, knowledge stock, which is stored in the brain of talents, working systems, and organizational culture, etc., provides the sources of innovation (Roper and Hewitt-Dundas, 2015; Caloghirou et al., 2018; Sung and Choi, 2018). Second, knowledge flow, which is driven by activities such as learning, sharing, and creation, enhances the capabilities of innovation (Roper and Hewitt-Dundas, 2015; Sung and Choi, 2018; Shi et al., 2020). Third, knowledge transfer and commercialization facilitate the realization of innovation performance (Ibidunni et al., 2020).

However, some scholars have argued that for enterprises, rather than knowledge stock or flow, the knowledge base is a more important factor to achieve their innovation goals based on the consideration of the following fact (Boh et al., 2014; Terjesen and Patel, 2017; Kobarg et al., 2019). It is impossible to stock all the knowledge required for innovation, so an enterprise needs to learn from the outside to obtain new one (Heide, 1994). Hence, a continuously growing firm needs to broadly search and adopt heterogeneous knowledge from environments, and integrate it with its existing deep knowledge (Lodh and Battaggion, 2015). Furthermore, from a structural perspective, Prabhu et al. (2005) proposed that depth and breadth constitute two dimensions of knowledge base, and provided a definition that knowledge breadth refers to the scope of knowledge possessed by enterprises, and that knowledge depth refers to the quantity of knowledge in the field of core technology.

Scholars have discussed the impact of knowledge base on innovation performance (Jin et al., 2015, 2018; Weng and Huang, 2017). They all have emphasized that the depth and breadth of knowledge can more accurately explain and predict the effect of knowledge than knowledge stock. The logic could be summarized as follows: deep knowledge in a fixed field supports firms to implement incremental innovation, enabling continuous product improvement and technological advantage in an industry (Triguero et al., 2018), and broad knowledge, through external search, brings firms more opportunities for radical innovation, promoting innovation performance in turbulent environments (Ye et al., 2019).

### Organizational Character for Innovation of Firm

The research on organizational character fell into three schools. First, scholars directly introduced individual personality theories (e.g., Big Five and MBTI) in psychology to explore the dimensions of organizational character (Costa and McCrae, 1992; Bridges, 2000). Second, other scholars argued that a firm does not have character originally, and so its character is the aggregation of personalities of employees (Slaughter et al., 2004). Finally, from a cognitive perspective, some scholars proposed that organizational character is the psychological reflection when stakeholders observe an organization and describe it in terms of human characteristics (Davies et al., 2004).

Organizational character exerts significant influences on the innovation of the firm. An open, foresighted, and risk-taking organization tends to implement an innovation strategy (Li and Yu, 2018; Zeb et al., 2021). On the contrary, a closed, myopic, and irresponsible one tends to avoid innovation risks (Li and Yu, 2018). A firm that is good at learning and sharing finds it easy to succeed in innovation, but a firm that lacks external and internal communication finds it difficult to create new things (Tamayo-Torres et al., 2016). In big data environments, digital personality and capability have become important enablers of massive, efficient, and sustainable innovation activities (Chatterjee et al., 2021; Zhen et al., 2021). Li and Yu (2018) and Xiao and Yu (2020) have assessed the positive effects of organizational character on innovation of firm.

## Development of Hypotheses

### The Mechanism of Innovation-Driven Growth

Survival and development are the eternal themes of enterprise management. Organizational growth makes a concentrated reflection of the two targets. According to the study of Resnick (2003), sustainable competitive advantage is supported by core competitiveness and differentiated competitiveness. The former helps enterprises to build their roots from scratch, and the latter helps enterprises to defeat their competitors and establish a brand of products. Consequently, the innovation of the firm should meet two-fold needs. For one thing, firms adapt to changes in external environments through innovation, thus building suitable ecological niches with their dynamic differentiation strategies (Ruiz-Moreno et al., 2016). For another, firms build core competitiveness through innovation, so as to provide sustainable power to support the development of organizations (Holahan et al., 2014). In practice, the enhancement of core competitiveness is usually supported by technological innovation, while business model innovation is often beneficial for the acquisition of temporary differentiated competitive advantage (Xiao and Yu, 2020).

Evidence for the relationship between technological innovation and organizational growth is abundant. Schumpeter (1912) suggested that technological innovation is to establish a new function of the combination of production factors, to obtain excess profits to a greater extent. Gifford (1992) found that in an industry, leading companies could locate the competition and establish a first-mover advantage through technological innovation. The overall optimization of innovation strategy is therefore beneficial to organizations to maximize the effectiveness of innovation and achieve a higher rate of economic growth. Taking sales growth rate as an observational variable, Uhlaner et al. (2013) explored the path of financial growth of an SME from a micro perspective and found that technological innovation plays a significant intermediary role. Bottazzi et al. (2001) confirmed the positive impact of technological innovation on organizational growth. Chen et al. (2012) further indicated that irrelevant technology innovation or diversification of innovation promotes innovation performance and organizational growth.

Business model design and marketing innovation play important roles in organizational growth (Osiyevskyy and Zargarzadeh, 2015). They help enterprises to form new business differentiation advantages in a short period of time, and weaken the defects of new entry, therefore obtaining competitive advantages and rapid growth (Zhang et al., 2017). Novelty-centered business model innovation enhances the attractiveness of products offered by enterprises and thereby directly improves organizational performance. Efficiency-centered business model innovation plays a regulatory role in the above-mentioned path and strengthens the impact of novelty-centered ones (Gerdoçi et al., 2018).

Technological innovation and business model innovation supplement and reinforce each other. According to the study of Ahlstrom (2010), all commercial activities are carried out to develop and popularize products and services, so an excellent enterprise should always bring innovation to the market, get resources from the market to feed innovation, and, finally, realize organizational growth through cyclic actions between market and innovation. As Camisón and Villar-López (2014) proposed, the complex mixture of technology, management, and market knowledge would induce different types of innovation, which improves the capabilities of enterprises with different structures and promote their growth through the development of comprehensive abilities. Similarly, Wei et al. (2014) suggested that exploratory and exploitative innovations generate different paths for organizational growth when they are supplemented by different types of business model innovation. Regardless of path, technological and business model innovations play a positive interactive role. According to Yun et al. (2015), the sustainable development of an SME relies on both knowledge-based technological and business model innovations, and both of which are indispensable.

Hence, we proposed the following hypotheses, and the relationships that we indicated constitute the mechanism of dual-innovation-driven growth.

H1: Technological innovation makes a positive impact on growth of firm.

H2: Business model innovation makes a positive impact on growth of firm.

H3: The interaction between technological and business model innovations reinforces the effects on growth of firm.

### The Impact of Knowledge Base on Innovation

Extensive knowledge search helps enterprises to explore, identify, and capture high-quality knowledge resources from an exterior environment. By accessing knowledge outside the boundaries, enterprises increase the diversity of knowledge portfolios, which would stimulate knowledge integration and collaborative creation. Carayannopoulos and Auster (2010) found that in highly competitive industries, companies are accustomed to the knowledge learned from other companies and implement R and D activities through imitation. When the knowledge domain becomes more complex, product innovation becomes more dependent on external knowledge acquisition. In addition, after the repeated emphasis on open innovation, some scholars have proposed that from the perspective of collaborative innovation, a broad source of knowledge would be conducive to cooperation, which may bring high innovation performance (Estrada et al., 2016; Grillitsch et al., 2017).

The development of deep knowledge encourages enterprises to better understand their technologies from a professional perspective. It lays the foundation for product improvement and process optimization. Focusing on specific research areas, enterprises can continuously deepen existing knowledge and generate new or more valuable knowledge. Since the integration of homologous knowledge can effectively reduce mismatches, problems between product development subsystems can be adequately addressed as knowledge depth increases (Jin et al., 2015). In addition, sufficient depth of knowledge means that enterprises have a competitive position in core technologies and products. The position can help enterprises determine the priorities of formulating technical standards for an industry, which support organizations to establish a positive feedback loop for the improvement of innovation capability (Prabhu et al., 2005).

A firm’s business model innovation relies on its ability to quickly collect and respond to external information, which is vital for the integration of external and internal resources. It does not require too many technological breakthroughs or product complexities. The simpler the product presentation is, the more likely a business model would be successful. Mina et al. (2014) analyzed some typical cases of business model innovation and found that successful companies have the following common features: they are good at searching widely for external knowledge and making full use of multi-source knowledge. In order to develop new product distribution channels, processes, and services, it is necessary to increase the diversification of internal and external resources. The capability of a broad external knowledge search contributes to the innovation of business models.

Hence, we proposed the following hypotheses:

H4: Knowledge base makes a positive impact on technological innovation.

H5: Knowledge base makes a positive impact on business model innovation.

### The Moderating Role of Organizational Character

Organizational character that reflects as culture, belief, value, norm, tradition, and routine, etc. (Moore, 2015), strengthens the awareness on innovation of employees. When employees are influenced by the above-mentioned factors, they would consider long-term development more, thus helping to spread awareness on the importance of innovation (Lin et al., 2011). In addition, the one whose individual personality fits with organizational character would be spiritually motivated to willingly share the risk of innovation-driven growth with a firm (Wu et al., 2016). According to the study of Ng et al. (2010), psychological contract and organizational commitment, representing psychological characteristics of organizations, play important roles in the induction process of technological innovation behaviors of employees.

Organizational character influences the logic and process of strategy design and institution generation (Moore, 2005). For one thing, strategies regarding technological innovation determine the allocation of resources and the development of capabilities that promote the transfer from knowledge to innovation achievements (Hussain and Terziovski, 2016). Led by a specific innovation strategy, knowledge-based innovation can play a better role and initiate innovation activities effectively. For another, institutions including resource management system, interest distribution mechanism, and intellectual property protection system constitute vital soft environments for collaborative technological innovation (Wieland, 2014). Furthermore, many scholars have demonstrated that organizational culture and ethics, equal to the organizational character in the study of Moore (2015), significantly strengthen innovation capability and increase technological innovation performance.

Organizational character affects product positioning and characteristics, which are related to the idea of technological innovation and product development. That is, the organizational character is connected with the logic and mechanism of innovation. It also dominates the way in which enterprises are providing products and services, since the direction and focus of innovation rely on the novel or efficient delivery methods.

Emerging enterprises that tend to pursue short-term benefits and have no belief in sustainable development may be more inclined to integrate and utilize existing knowledge and even rely on external knowledge to promote novelty-centered business model innovation, thereby gaining competitive advantages by capturing casual market opportunities. On the contrary, a stable enterprise pursuing sustainable development and growth would pay more attention to the positive role of technological innovation and make long-term arrangements for its technological strategy. Moreover, when developing business models, the enterprise would think more about value chain planning and strategic market layout and consider the integration and utilization of knowledge from a macro perspective.

Hence, we proposed the following hypotheses:

H6: Organizational character positively moderates the impact of knowledge base on technological innovation.

H7: Organizational character positively moderates the impact of knowledge base on business model innovation.

### Research Framework

**Figure 1:**
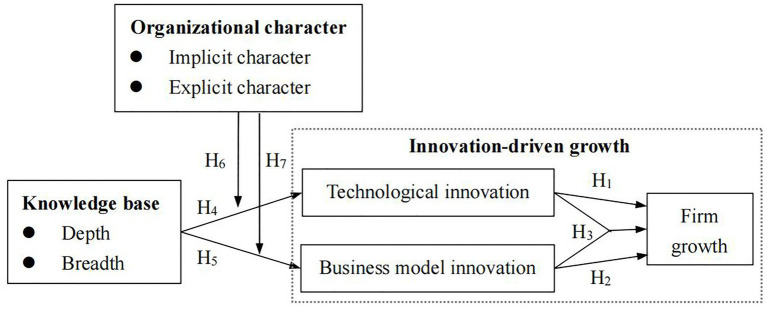
The framework of this study.

According to the analyses mentioned above, we proposed the research framework, as shown in Figure 1. In this framework, we think of innovation-driven growth as a whole and then focus on the assessment of the direct effects of knowledge base and the moderating effects of organizational character on innovation-driven growth but neglect to assess the mediating role of innovation in the relationship between knowledge base and growth of the firm. The setting is beneficial to realize our research targets: improving theories and getting new insights regarding firm dual-innovation-driven growth supported by the collaborative governance between the knowledge base and organizational character.

## Methodology

### Sampling and Data Collection

Considering the availability of data, the sample was determined to be companies on Shanghai Stock Exchange in China listed before 2019. Considering the possible time lag of the innovation effect, the dependent variable, which is organizational growth, was measured on the basis of 2017 data, and for the measurement of other variables, the data from 2016 was used.

Sample selection followed the following criteria: first, companies should have been listed for at least 2 years to ensure access to public data from 2016 and 2017. Second, taking into account the data comparability of variables such as innovation and growth, companies in the real estate, education, and financial industries were deleted in accordance with the newly issued listed company industry of China Securities Regulatory Commission Classification. The listed companies that were specially treated (ST) by China Securities Regulatory Commission because of two consecutive years of losses and listed companies with incomplete data were also deleted.

After screening, the study finally retained the data of 965 listed companies. Sample enterprises were distributed in a wide range of industries, including the mining industry that accounts for 5.1%; the electricity, heat, gas, and water production, and supply industries that account for 6%; the construction industry that accounts for 4.5%; the transportation, warehousing, and postal industries that account for 6.3%; the technical services that account for 1.5%; the agriculture, forestry, animal husbandry, and fishery industries that account for 1.6%; the water conservancy, environment, and public facilities management industry that accounts for 1.1%; the information transmission, software, and information technology services that account for 5.5%; the manufacturing industry that accounts for 67%; and finally the comprehensive industry that accounts for 1.5%. In terms of employee quantity, 22.5% of the sample enterprises have less than 1,000 employees; 45.1% has employees between 1,000 and 5,000; 15.5% has employees between 5,001 and 10,000; 14.3% has employees between 10,001 and 50,000; and, finally, 2.6% of the enterprises have more than 50,000 employees. As for sample distribution according to age, 3.4% of the enterprises are less than 10 years old, 49.7% is between 10 and 20 years old, 45% is between 20 and 30 years old, and 1.9% is above 30 years old.

### Measures of Construct

Four constructs, i.e., knowledge base, organizational character, innovation, and growth of firm were developed to test the proposed hypotheses. To better control the biases of quantitative analysis, we developed several control variables. In order to avoid the subjective biases of survey data collected by reflective scales, we chose to develop quantitative indicators to assess the latent constructs. This method can help us to collect and use the public data that were reported by Chinese listed companies. It improved the feasibility of the assessment and the quality of the research data.

#### Knowledge Base

Learning from Ozman (2007) and Xu (2015), we adopted the patent data based on international patent classification (IPC) coding, and the statistical variables to assess knowledge breadth and depth. Although the data of patent cannot reflect knowledge base since some other types of knowledge, such as technological secret and tacit knowledge, patent data were still widely used in empirical studies to represent knowledge base because of the advantages of objectivity and availability of data (Jiang et al., 2020; Vakili and Kaplan, 2021). According to IPC patent classification, the number of classes of authorized patents for a firm was used to represent knowledge breadth. A firm may develop different amounts of patents in different classes. When a firm develops the largest number of patents in a class, we regarded it as the main domain of its technological knowledge. We, therefore, set the maximum number of patents in a certain class as knowledge depth. Considering the differences in different firms, we developed ratio-type indicators to assess the knowledge base. In detail, the formulas were presented in Table 1.

**Table 1:** Variables and indices.

Concepts	Variables	Abbr.	Description	Measure or index
Knowledge Base	Knowledge Breadth	KB	Domain scope of knowledge owned by enterprises	$KB_{j}=\frac{b_{j}}{d_{j}}$^a^
	Knowledge Depth	KD	The amount of knowledge in the domain of enterprise knowledge	$KD_{j}=\frac{d_{j}}{N_{j}}$^a^
Organizational Character	Implicit character	IC	Agreeableness	Employee pay payable/Operation revenue^c^
			Neuroticism	Current ratio
	Explicit Character	EC	Conscientiousness	Is social responsibility report open to the public?^e^
			Extroversion	Selling expenses/Operation revenue
			Openness	OFDI/Ownership rights
Innovation	Technological Innovation	TI	Cost of technology introduction and learning	Purchase costs of patents, proprietary technologies and non-proprietary technologies
			Income from the transfer and licensing of patents	Disposal costs of patents, proprietary technologies and non-proprietary technologies
			Product innovation	Are new products developed?^e^
			Process innovation	Are production processes introduced or improved?^e^
	Business Model Innovation	BMI	Activities that improve transaction efficiency or reduce transaction costs	Is there an online trading platform?^e^
				Is there a perfect logistics system?^e^
			Activities to develop new trading methods or expand trading networks	Is there a customized service?^e^
				Does it introduce new partners?^e^
Firm growth		OG	Changes of corporate income	Growth rate of operating profit
				Growth rate of operating revenue
			Changes of corporate assets	Growth rate of net asset value per share
				Growth rate of total assets turnover

a *b_j_* indicates the number of IPC patent classes involved with the authorized patents of the company j in 2016. *d_j_* indicates the maximum number of authorized patent in each class for the company *j* in 2016. *N_j_* is the total number of patents of the company *j* in 2016.

c Employee pay payable includes wage, bonus, allowance, and subsidy.

e Dummy variables, No = 0, Yes = 1.

#### Organizational Character

The Big Five theory guided us in designing the variables of organizational character (Geuens et al., 2009; Kang et al., 2016). Among the five dimensions of organizational character, agreeableness and neuroticism were classified as an implicit character, and conscientiousness, extroversion, and openness were classified as an explicit character. We developed instrumental indicators to represent the dimensions of organizational character so as to collect public and financial data of targeted firms. First, we determine the ratio of employee pay payable to operation revenue, which significantly influences the perception of happiness of employees, to assess the agreeableness of a firm. Second, we used the current ratio to reversely reflect the neuroticism of a firm, since it would be perceived a risk when the cash flow of a firm is short. Third, only few Chinese listed companies will publish their social responsibility reports, so we assessed the conscientiousness of a firm by investigating whether its report is open to the public. Fourth, a firm can better manage its external relational capital when it invests more in market development; thus, we used the ratio of selling expenses to operating revenue to assess extroversion. Finally, we determine the ratio of outward foreign direct investment to ownership rights to assess openness.

#### Innovation

The process of technological innovation includes knowledge creation and technology transfer. With reference to the research of Yu et al. (2017) and Azar and Ciabuschi (2017), knowledge creation was investigated by the purchasing expenses and disposal earnings of patented technologies, and technology transfer was assessed from the perspective of product and process innovations. The two were, respectively, assessed by one item “are new products developed” and “are production processes introduced or improved,” since their quantifiable data could not be collected conveniently. According to Zott and Amit (2008), business model innovation was divided into efficiency-centered and novelty-centered business models. Likewise, we, respectively, developed two true-false items to assess them. The items of efficiency-centered one include “is there an online trading platform” and “is there a perfect logistics system,” and; those of novelty-centered one include “is there a customized service” and “does it introduce new partners.” These items were adapted from the study of Zott and Amit (2008). The authors could well mark these items after collecting adequate information about targeted firms. All members of the research team jointly gave scores on the basis of negotiation and training.

#### Growth of Firm

In practice, the growth of the firm often reflects changes in income and assets (Penrose, 2002), which, respectively, represent growth condition and growth quality. We assessed income change by the growth rate of operating profit and the growth rate of operating revenue. Asset change was assessed by the growth rate of net asset value per share and the growth rate of total assets turnover.

#### Control Variables

Learning from previous studies (Azar and Ciabuschi, 2017; Snihur and Wiklund, 2019), we designed four control variables. (1) Logarithm of size. The size of an enterprise is represented by the number of employees in it. The logarithm method is used to reduce the impact of extreme values. (2) Age. From the time a sample company was established to 2018. (3) Industry. It is a dummy variable. A non-manufacturing industry was coded as 0, and a manufacturing industry was coded as 1. (4) Ownership. It is also a dummy variable. A state-owned enterprise (including municipal state-owned, provincial state-owned, and central state-owned enterprises) was coded as 0, and a non-state-owned enterprise (including collective, private, and foreign-funded enterprises, and others) was coded as 1.

The detail of measures development can be seen in Table 1. The methods to calculate the constitutive data of the constructs in this study are as follows: first, we directly added the values of the four items to obtain the variable data of business model innovation (round numbers range from 0 to 4). Second, for the explicit character, technological innovation and firm growth, principal component analysis was conducted to calculate the weights, and the data of principal components were used for measurement. However, *the* implicit character was exceptional. This variable directly averaged the data of two indicators since only two observational indices were not applicable to principal component analysis. In addition, different from the reflection scale, the constitutive scale in our study, which is composed of instrumental indicators, does not need for its reliability and validity to be assessed.

### Descriptive Statistics

Table 2 displays the mean values, standard deviations, and correlation coefficients for core variables. It is found that knowledge breadth is significant positively correlated with technological innovation (*ρ* = 0.126, *p* < 0.01) and business model innovation (*ρ* = 0.196, *p* < 0.01). Knowledge depth is significant positively correlated with technological innovation (*ρ* = 0.214, *p* < 0.01) and business model innovation (*ρ* = 0.211, *p* < 0.01). Implicit character is significant negatively correlated with business model innovation (*ρ* = −0.101, *p* < 0.01). Explicit character is significant negatively correlated with technological innovation (*ρ* = −0.092, *p* < 0.01) but significant positively correlated with business model innovation (*ρ* = 0.270, *p* < 0.01). Technological innovation is significant positively correlated with business model innovation (*ρ* = 0.071, *p* < 0.05) and organizational growth (*ρ* = 0.528, *p* < 0.01). Finally, business model innovation is also significant positively correlated with organizational growth (*ρ* = 0.130, *p* < 0.01). In a word, significant correlation relationships do exist between the core variables involved in this study, and most of them are positive. This provides preliminary evidence for the research hypotheses.

**Table 2:** Means, SD, and correlation coefficients.

Variables	Mean	SD	1	2	3	4	5	6	7	8	9	10	11
Ln(Size)	7.958	1.427	1										
Age	20.420	4.968	−0.072^**^	1									
Industry	0.670	0.470	−0.026	0.004	1								
Ownership	0.510	0.500	−0.299^***^	−0.036	0.239^***^	1							
KB	0.598	0.815	−0.078^**^	−0.170^***^	0.132^***^	0.035	1						
KD	0.400	0.406	−0.057	−0.163^***^	0.208^***^	0.125^***^	0.329^***^	1					
IC	1.841	1.555	−0.250^***^	0.100^***^	0.000	0.250^***^	0.020	0.045	1				
EC	10.650	14.237	0.250^***^	0.113^***^	−0.226^***^	−0.334^***^	0.016	−0.068^***^	−0.186^***^	1			
TI	0.504	0.155	0.055	−0.073^**^	0.397^***^	0.172^***^	0.126^***^	0.214^***^	0.058	−0.092^***^	1		
BMI	1.830	1.095	0.191^***^	−0.020	0.043	−0.091^***^	0.196^***^	0.211^***^	−0.101^***^	0.270^***^	0.071^**^	1	
OG	3.390	3.892	0.088^***^	−0.053	0.394^***^	0.088^***^	0.113^***^	0.171^***^	0.015	−0.068^**^	0.528^***^	0.130^***^	1

*N* = 965.

** *p* < 0.05;

*** *p* < 0.01 (two-tailed tests).

### Methods

Structural equation model is not suitable for the data analysis of this study because of the following reasons: first, the independent variables of the study were, respectively, measured by a single statistical indicator rather than several reflective items. Second, the numbers of instrumental indicators of several constructs are less than three, restricting the estimation of coefficients by structural equation modeling. Finally, in this study, instrumental indicators constitute but do not reflect constructs. Conventional structural equation models are not convenient to deal with constitutive data.

In order to reduce the possible impact of multi-collinearity, all data were centralized, and the values of interaction terms were also calculated based on centralized data. After processing, multi-collinearity was effectively controlled. In addition, the sample number of the study has well met the requirement for model estimation. Hence, partial least squares method is not indispensable.

We, therefore, constructed regression analysis models to test the hypotheses. First, in order to better display the effect of an independent variable, we gave priority to the hierarchical regression analysis method in our data analysis processes. *p* value was taken as a criterion for the hypothesis test. When the *p* value was smaller than 0.1, we chose to accept the null hypothesis that was proposed in the research framework. We reported the significance of *F* value, *R*-square, and adjusted *R*-square to comprehensively reflect the quality of regression models. Moreover, the maximum value of variance inflation factor was used for collinearity diagnostics. Its recommended value is less than 10.

Second, because business model innovation, which is one of the dependent variables of the study, is ordinal, ordered multiple classification logistic regression was used to test the effect of knowledge base on business model innovation. When the results of ordered multiple classification logistic regression analysis could not pass the parallel lines test, the binary logistic regression analysis method replaced it. In logistic regression analysis models, likewise, the *p* value was used to judge whether a hypothesis should be accepted or refused. Different from the hierarchical regression analysis method, model fitting information with respect to −2 log likelihood value, chi-square, df, and sig. was used to display the effectiveness of model fitting. The results of the Pearson test and deviance test assisted us to judge for the goodness of fit. In contrast, in binary logistic regression analysis models, the goodness of fit was assessed by the omnibus test and Hosmer-Lemeshow test. Test of parallel lines should be passed when ordered multiple-classification logistic regression model is used. The recommended sig. value in the test of parallel lines is greater than 0.05. Cox and Snell’s method was used to provide the pseudo-R-square of the whole logistic regression analysis model.

Interactive items were introduced into all of the models to assess the moderating effect of organizational character.

SPSS 20 (IBM, New York) software tools were used for statistical and quantitative analyses of data.

## Results

### The Effects of Innovation on Growth of Firm

The fitting effects of technological and business model innovations on organizational growth are shown in Table 3. According to the results of models M1–M4, both technological (*β* = 0.438, *p* < 0.01) and business model innovations (*β* = 0.099, *p* < 0.01) have significant effects on growth of firm, but the effect of technological innovation is much more intense. In addition, the interaction effect (*β* = 0.112, *p* < 0.01) is also significant. Therefore, H1, H2, and H3 all passed the test.

**Table 3:** Hierarchical regression analysis results of firm growth.

Variances		M1	M2	M3	M4
Control variances	Ln(Size)	0.102^***^	0.061^**^	0.084^***^	0.05^*^
	Age	−0.046	−0.018	−0.045	−0.020
	Industry	0.391^***^	0.227^***^	0.385^***^	0.230^***^
	Ownership	0.023	−0.024	0.029	−0.017
Independent variances	TI		0.438^***^		0.438^***^
	BMI			0.099^***^	0.076^***^
Interaction terms	TI*BMI				0.112^***^
Goodness of fit	F	48.277^***^	92.353^***^	41.230^***^	71.478^***^
	R^2^	0.409	0.570	0.421	0.586
	Adj R^2^	0.164	0.321	0.173	0.339
	Maximum VIF	1.171	1.233	1.174	1.238

*N* = 965.

* *p* < 0.10;

** *p* < 0.05;

*** *p* < 0.01.

### The Effects on Technological Innovation

Taking technological innovation as the dependent variable, and knowledge base and its interaction terms with organizational character as independent variables, the study used the method of hierarchical regression analysis to assess the impacts of knowledge base and organizational character on technological innovation. The results are shown in Table 4.

**Table 4:** Hierarchical regression analysis results of technological innovation.

Variances		M5	M6	M7	M8	M9	M10	M11	M12	M13
Control variances	Ln(Size)	0.093^***^	0.088^***^	0.083^***^	0.082^***^	0.088^***^	0.089^***^	0.087^***^	0.083^***^	0.095^***^
	Age	−0.064^**^	−0.054^**^	−0.046	−0.042	−0.053^*^	−0.054^*^	−0.047	−0.046	−0.039
	Industry	0.374^***^	0.367^***^	0.352^***^	0.350^***^	0.367^***^	0.367^***^	0.350^***^	0.352^***^	0.354^***^
	Ownership	0.108^***^	0.107^***^	0.096^***^	0.097^***^	0.106^***^	0.107^***^	0.100^***^	0.096^***^	0.092^***^
Independent variances	KB		0.058^*^		0.027	0.057^*^	0.057^*^			0.022
	KD			0.117^***^	0.109^***^			0.116^***^	0.117^***^	0.109^***^
Interaction terms	KB*IC					0.008				0.030
	KB*EC						−0.008			−0.006
	KD*IC							−0.050^*^		−0.061^*^
	KD*EC								0.009	0.003
Goodness of fit	F	51.596^***^	42.139^***^	44.837^***^	37.481^***^	35.295^***^	35.096^***^	37.935^***^	37.346^***^	19.336^***^
	R^2^	0.421	0.424	0.435	0.436	0.424	0.424	0.438	0.435	0.443
	Adj R^2^	0.174	0.176	0.185	0.185	0.175	0.175	0.187	0.184	0.186
	Maximum VIF	1.171	1.172	1.182	1.186	1.178	1.172	1.187	1.182	1.312

*N* = 965.

* *p* < 0.10;

** *p* < 0.05;

*** *p* < 0.01.

The results of M5–M8 show that both knowledge breadth (*β* = 0.058, *p* < 0.1) and knowledge depth (*β* = 0.117, *p* < 0.01) have significant positive effects on technological innovation, but the effect of knowledge depth is more significant and stronger than that of knowledge breadth. That is, H4 has passed the test. However, when both of them work together on technological innovation, the effects of knowledge depth (*β* = 0.109, *p* < 0.01) and knowledge breadth (*β* = 0.027, *p* > 0.1) are all reduced, and the adjusted R^2^ of model M8 is not significantly improved compared with that of model M7. It indicates that when a firm continuously implements technological innovation at a high level of knowledge depth, it is hard for knowledge breadth to make marginal contributions.

The results of M9–M13 show that organizational character could hardly positively moderate the relationships between knowledge base and technological innovation. M11 even shows that implicit character (*β* = −0.05, *p* < 0.1) can negatively moderate the positive impact of knowledge depth on technological innovation. M13 shows that knowledge depth is the core influencing factor of technological innovation, and that the organizational investment in other intangible resources would inhibit the positive effect of knowledge depth. Hence, H6 could not pass the test.

### The Effects on Business Model Innovation

Since business model innovation is a categorical variable in order, we adopted the logistic regression model of ordered multiple-classification for hypothesis test, and the analysis results are shown in Table 5.

**Table 5:** Results of ordered multiple-classification logistic regression analysis of business model innovation.

Variances		M14	M15	M16	M17	M18	M19	M20	M21	M22
Control variances	Size	0.335^***^	0.315^***^	0.317^***^	0.306^***^	0.316^***^	0.306^***^	0.321^***^	0.313^***^	0.182^***^
	Age	−0.007	0.047	0.054	0.083	0.042	0.044	0.053	0.052	0.001
	Industry	0.108^*^	0.059	0.036	0.013	0.053	0.055	0.033	0.029	0.094
	Ownership	−0.084	−0.096	−0.131^**^	−0.130^**^	−0.089	−0.098	−0.126^**^	−0.132^**^	−0.011
Independent variances	KB		0.348^***^		0.251^***^	0.355^***^	0.358^***^			0.251^***^
	KD			0.387^***^	0.315^***^			0.386^***^	0.395^***^	0.300^***^
	IC									−0.079
	EC									0.545^***^
Interaction terms	KB*IC					−0.086				−0.014
	KB*EC						0.151^**^			0.141^*^
	KD*IC							−0.061		0.002
	KD*EC								0.112^*^	0.088
Goodness of fit	−2 Log Likehood value	2797.518	2767.054	2758.731	2743.671	2764.972	271.759	2757.831	2755.560	2671.687
	Pearson test (Chi-Square, df and sig. respectively)	40.394 4 <0.01	70.858 5 <0.01	79.181 5 <0.01	94.241 6 <0.01	72.939 6 <0.01	76.153 6 <0.01	80.080 6 <0.01	82.351 6 <0.01	166.225 12 <0.01
	Deviance test (Chi-Square, df and sig. respectively)	3835.949 3,848 > 0.05	3806.755 3,847 > 0.05	3799.840 3,847 > 0.05	3781.165 3,846 > 0.05	3804.339 3,850 > 0.05	3820.389 3,850 > 0.05	3788.191 3,850 > 0.05	3811.638 3,850 > 0.05	3825.553 3,844 > 0.05
	Test of Parallel Lines (sig.)	0.580	0.326	0.076	0.045	0.370	0.214	0.006	0.110	0.002
	Cox and Snell	0.041	0.071	0.079	0.093	0.073	0.076	0.080	0.082	0.158

*N* = 965.

* *p* < 0.10;

** *p* < 0.05;

*** *p* < 0.01.

The results of models M15 and M16 show that both knowledge breadth (*β* = 0.348, *p* < 0.01) and knowledge depth (*β* = 0.387, *p* < 0.01) have significant positive effects on business model innovation. According to model M17, when both of them work together on business model innovation, knowledge depth (*β* = 0.315, *p* < 0.01) plays a more significant role than knowledge breadth (*β* = 0.251, *p* < 0.01). However, the parallel line test of M17 could not be passed, indicating that the results may not be credible. Considering that the data of business model innovation is accumulated by several observational variables, the order of the categorical variable may be destructively affected to some extent. Therefore, the fitting method was changed to binary logistic regression analysis, and the result (M23) is shown in Table 6. M23, which has passed the Hosmer-Lemeshow test, still supports the existing conclusion. In summary, H5 passed the test.

**Table 6:** Binary logistic regression analysis of business model innovation.

Variances		M23	M24	M25
Control variances	Ln(Size)	0.297^***^	0.310^**^	0.158^*^
	Age	0.030	−0.011	−0.057
	Industry	−0.090	−0.073	−0.019
	Ownership	−0.109	−0.101	0.048
Independent variances	KB	0.273^***^		0.255^***^
	KD	0.434^***^	0.482^***^	0.436^***^
	IC			−0.002
	EC			0.580^***^
Interaction terms	KB*IC			−0.025
	KB*EC			0.230^**^
	KD*IC		−0.099	−0.045
	KD*EC			−0.012
Goodness of fit	Omnibus test of model coefficients (Chi-Square, df and sig. respectively)	73.103 6 <0.05	61.913 6 <0.05	122.007 12 <0.05
	Hosmer-Lemeshow test (Chi-Square, df and sig. respectively)	10.268 8 0.247>0.05	8.384 8 0.397>0.05	4.299 8 0.829>0.05
	−2 Log Likehood value	1007.232	1018.422	958.328
	Cox and Snell	0.073	0.062	0.119

*N* = 965. Business model innovation after binary classification processing (0/1) is the dependent variable. The binary classification processing rule is as follows: when the initial business model innovation >=3, the value is 1; otherwise, it is 0.

* *p* < 0.10;

** *p* < 0.05;

*** *p* < 0.01.

The results of models M19 and M21 show that explicit character significantly moderates the positive impact of knowledge breadth (*β* = 0.151, *p* < 0.05) and knowledge depth (*β* = 0.112, *p* < 0.1) on business model innovation. According to the results of models M18 and M20, the implicit character has no significant moderating effect on the path of business model innovation influenced by the knowledge base. Since the parallel line test of M20 could not be passed, the binary logistic regression analysis was alternatively used, and the result is shown as M24 in Table 6. However, the result still does not support the significant moderating effect of implicit character. Therefore, H7 partly passed the test.

The regression of saturated model (M22) could not pass the parallel line test, and the alternative results by binary logistic regression analysis are shown as M25 in Table 6. The results show that business model innovation can be really attributed to knowledge base and organizational character, and their interaction. Knowledge depth (*β* = 0.436, *p* < 0.01) and knowledge breadth (*β* = 0.255, *p* < 0.01) have positive impacts on business model innovation. Explicit character (*β* = 0.58, *p* < 0.01) also contributed significantly to it. Moreover, explicit character significantly strengthens (*β* = 0.23, *p* < 0.05) the positive effect of knowledge breadth on business model innovation. However, the moderating effect of explicit character on the path of business model innovation influenced by knowledge depth is not significant. In addition, for business model innovation, the direct and indirect effects of implicit character are all not significant. The results repeatedly confirm the conclusions of hypotheses H5 and H7.

All in all, we summarized our research results and conclusions of the hypotheses test in Table 7.

**Table 7:** Summary of the results.

No.	Hypotheses	Decisions
H1	Technological innovation <-- Firm growth	Accepted
H2	Business model innovation <-- Firm growth	Accepted
H3	Technological innovation × Business model innovation <-- Firm growth	Accepted
H4	Knowledge base <-- Technological innovation	Accepted
	Knowledge breadth <-- Technological innovation	Accepted
	Knowledge depth <-- Technological innovation	Accepted
H5	Knowledge base <-- Business model innovation	Accepted
	Knowledge breadth <-- Business model innovation	Accepted
	Knowledge depth <-- Business model innovation	Accepted
H6	Knowledge base × Organizational character <-- Technological innovation	Refused
	Knowledge breadth × Implicit character <-- Technological innovation	Refused
	Knowledge breadth × Explicit character <-- Technological innovation	Refused
	Knowledge depth × Implicit character <-- Technological innovation	Refused
	Knowledge depth × Explicit character <-- Technological innovation	Refused
H7	Knowledge base × Organizational character <-- Business model innovation	Partly accepted
	Knowledge breadth × Implicit character <-- Business model innovation	Refused
	Knowledge breadth × Explicit character <-- Business model innovation	Accepted
	Knowledge depth × Implicit character <-- Business model innovation	Refused
	Knowledge depth × Explicit character <-- Business model innovation	Accepted

## Conclusion, Implications, and Limitations

### Conclusion

In the study, we proposed a new framework for explaining the mechanism of innovation-driven growth of the firm. Our contribution mainly reflects in the development of new theoretical thoughts, such as collaborative governance between the knowledge base and organizational character, and technological and business model innovations based on empirical analyses. Though the study was proposed and demonstrated on basis of the data of Chinese enterprises, its logic, framework, and findings are general. The main conclusions of the study were summarized as follows: first, similar to some previous ones (Hu, 2012; Rantala et al., 2018; Zhou et al., 2019; Xiao and Yu, 2020), this study proposed a theoretical framework for the growth of the firm, which is driven by both technological and business model innovations. However, different from the co-evolution relationship of technological and business model innovations that was proposed before (Hu, 2012; Zhou et al., 2019), a partial shared governance model would be constructed to promote innovation-driven growth of the firm, since our study found that (1) both technological and business model innovations have significant impacts on the growth of firm; (2) their interaction further strengthens the effect; but (3) the direct effect of technological innovation and the interactive effect are much greater than the direct effect of business model innovation. The partial shared governance model indicates that firms need to build a dual innovation system for collaboratively governing technological and business model innovations, but they should give priority to the development of technological innovation.

Second, unlike the study of Xiao and Yu (2020), which paid attention to the assessment of mediating effects of technological and business model innovations on the relationship between knowledge, character, and sustainable competitive advantage, this study considered innovation and growth as a whole, and then focused on assessing the interactive effect of knowledge structure and organizational character on innovation-driven growth. Through this action, we obtained three important findings: (1) to promote technological innovation, knowledge base almost makes the total contribution; (2) knowledge base and organizational character work together to facilitate business model innovation; (3) the combination of knowledge base and explicit character constitutes a good strategy to accelerate business model innovation. Enlightened by the findings, we proposed a new idea of the collaborative governance between the knowledge base and organizational character. The collaborative governance emphasizes two key things: (1) firms should cultivate their explicit characters; and (2) they should match their explicit characters with the knowledge base for balanced and co-evolutionary development. The idea contributes to the development of innovation-driven growth theory.

Finally, different from prior studies that paid attention to the roles of knowledge stock or knowledge flow (Roper and Hewitt-Dundas, 2015; Caloghirou et al., 2018; Sung and Choi, 2018; Shi et al., 2020), this study assessed the effects of the knowledge base (depth and breadth). The ambidexterity theory would help us better understand the relationships between knowledge depth and breadth. In the study, we obtained three findings: (1) technological innovation relies more on knowledge depth than breadth; (2) knowledge depth and breadth jointly drive business model innovation; and (3) explicit character could significantly strengthen the effect of knowledge breadth on business model innovation. These findings contribute to the development of innovation theory from a knowledge-based view.

### Theoretical and Practical Implications

This study could enlighten researchers on four aspects. First, a partial shared governance model for innovation-driven growth was appealed for deep study in the future. This study led a new perspective to consider the relationships between technological and business model innovations. Second, we reinforced the roles of organizational character. In fact, most previous studies on the organizational character were conceptual. This study introduced it into the explanation and prediction of innovation-driven growth. It may trigger more studies about organizational character. Third, the idea of collaboratively governing knowledge base and organizational character would be an upgrade for intangible resource management. It arouses scholars to rethink the components of firm core intangible assets, the governance of which is conducive to higher efficiency. Finally, the heterogeneous effects of knowledge depth and breadth remind us to deeply explore the structure of corporate knowledge assets. For resource utilization efficiency, the structure should be as important as stock and flow in many cases. It is essential to construct an ambidextrous system to manage the depth and breadth of knowledge.

This study gives practitioners three managerial implications. First, managers need to build dual innovation systems for their firms. In the system, technological innovation provides a core impulse for the growth of the firm. They surely should not neglect the importance of business model innovation. Second, they should reinforce the management of intangible resources, especially knowledge base and organizational character. Collaborative governance is an effective strategy that we recommend. Third, they are aroused to well match the resources, innovation behaviors, and growth strategy of firms. For an endogenous growth strategy, managers should pay attention to the enhancement of technological innovation capability. The center of management is continuous knowledge development in the corporate core technology field. For an exogenous growth strategy, managers need to transfer their attention from technological innovation to business model innovation. In order to facilitate business model innovation, they should well balance knowledge depth and breadth, and well match knowledge base and explicit character.

### Limitations and Future Research

Some limitations do exist in this study. First, measured deviations and subjective biases may generate when we took instrumental indicators and quantitative data to measure latent variables. The effectiveness of independently developed instrumental variables needs more examination. Second, some of our findings may vary across different cultures. The robustness of our research results needs further tests. Finally, the mediating effects of innovation on the relationship between the knowledge base and firm growth were neglected to refine the research framework. However, they do exist.

In the future, we will plan to improve our study from two aspects. First, we will redesign the measurement scale and re-demonstrate the framework on the basis of a questionnaire survey of large samples. Second, we will hope to carry out global cooperative research, through which the findings could be generally verified. Finally, we will continuously improve the study framework through the combination of thinking of practical experience and theoretical logic.

## Data Availability Statement

The raw data supporting the conclusions of this article will be made available by the authors, without undue reservation.

## Author Contributions

DY proposed the idea, designed the research, revised and checked the manuscript. HY collected and analyzed the data, and wrote the draft manuscript. Both the authors contributed to the article and approved the submitted version.

### Conflict of Interest

The authors declare that the research was conducted in the absence of any commercial or financial relationships that could be construed as a potential conflict of interest.
